# Tunnelled dialysis catheter tract haemorrhage during mechanical thrombectomy of right atrial line-associated thrombus and line removal: a case report

**DOI:** 10.1186/s42155-025-00610-6

**Published:** 2025-10-28

**Authors:** Ivan Jobling, Johnny O’Mahony, Benedict Thomson, Nick Ware, Narayan Karunanithy, Narayanan Thulasidasan, Athanasios Diamantopoulos

**Affiliations:** 1 Department of Interventional Radiology, Guy’s and St., Thomas’ NHS Trust, St Thomas’ Hospital, 1st Floor Lambeth Wing, Westminster Bridge Road, London, SE1 7EH UK; 2 School of Biomedical Engineering & Imaging Sciences, Faculty of Life Sciences & Medicine, Kings College London, Westminster Bridge Road, London, SE1 7EH UK; 3 Clinical Imaging and Medical Physics, Guy’s and St Thomas, 1st Floor Lambeth Wing, Westminster Bridge Road, SE1 7EH London, UK

**Keywords:** Thrombectomy, Haemorrhage, Right atrial thrombus, Paediatric, Interventional

## Abstract

**Background:**

Central venous access catheter (CVC) devices are a critical part of care in patients with a variety of treatment needs but are not without complications.

**Case presentation:**

A 5-year-old male child with a background of autosomal recessive nephrotic syndrome developed a septic right atrial thrombus related to his dialysis line. Mechanical thrombectomy and line removal was complicated by tract haemorrhage, requiring the integrated management of thrombosis and haemorrhage. Haemostasis was achieved with combination of compression and sealing of the subcutaneous tract.

**Conclusion:**

This case provides an excellent example of tract haemorrhage demonstrated fluoroscopically. We discuss the available treatment options, which may need to be employed in urgent or emergent fashion, in paediatric patients with differing physiological reserves.

## Introduction

Complications associated with CVC insertion and removal are well known, including vessel injury/stenosis/thrombosis, occlusion/malfunction/fracture and infection [1, 2].

More rarely, CVC can function as a nidus for the formation of thrombi in the right atrium leading to severe complications, including pulmonary embolism or life-threatening cardiac dysfunction. If the thrombus is infected it can also lead to endocarditis, septic emboli and septic shock [3]. Although no randomised control trials exist, previous analysis of septic right atrial thrombosis in adults has shown fewer serious complications post thrombectomy compared to medical treatment alone [3]. It is also an important adjunct when medical management with anticoagulation is insufficient or contraindicated.

However, this procedure also carries significant risks compared to systemic treatment. Tract haemorrhage after line removal is an uncommon but potentially dangerous event, particularly in paediatric patients, who have a relatively low circulating volume compared to adults and different thresholds for blood transfusion [4, 5].

This case report describes a 5-year-old boy who developed tract haemorrhage after central line removal following a successful right atrial mechanical thrombectomy. We discuss the management options available in the interventional suite and the importance of recognition and control of this complication in this patient population.

## Case report

### Patient history

The patient was a 5-year-old boy attending the paediatric nephrology service with renal failure secondary to recurrent autosomal recessive nephrotic syndrome after transplant. A month prior to his admission, a left internal jugular vein tunnelled dialysis catheter (14.5Fr, 23 cm catheter), was placed to facilitate ongoing plasmapheresis. During attendance for dialysis the patient was found to be pyrexial with rigors and was admitted for treatment with intravenous antibiotics. The patients tunnelled line became blocked preventing his next dialysis treatment. Echocardiogram performed showing a 2.0 × 1.4 cm thrombus within the right atrium related to the catheter.

Following multidisciplinary team (MDT) discussion between interventional radiology, paediatric cardiology and paediatric nephrology teams the decision was made not to remove the catheter, primarily due to the risk of septic emboli. This was a particular concern as the patient was immunosuppressed following his renal transplant.

Medical management with treatment dose dalteparin was unsuccessful with no underlying coagulopathy detected. A repeat echocardiogram (4 days later) demonstrated a larger catheter associated right atrial thrombus. The decision was made to proceed with mechanical thrombectomy followed by line removal.

### Management

Procedure was performed in dedicated paediatric interventional suite under general anaesthetic in supine position.

**Fig. 1:**
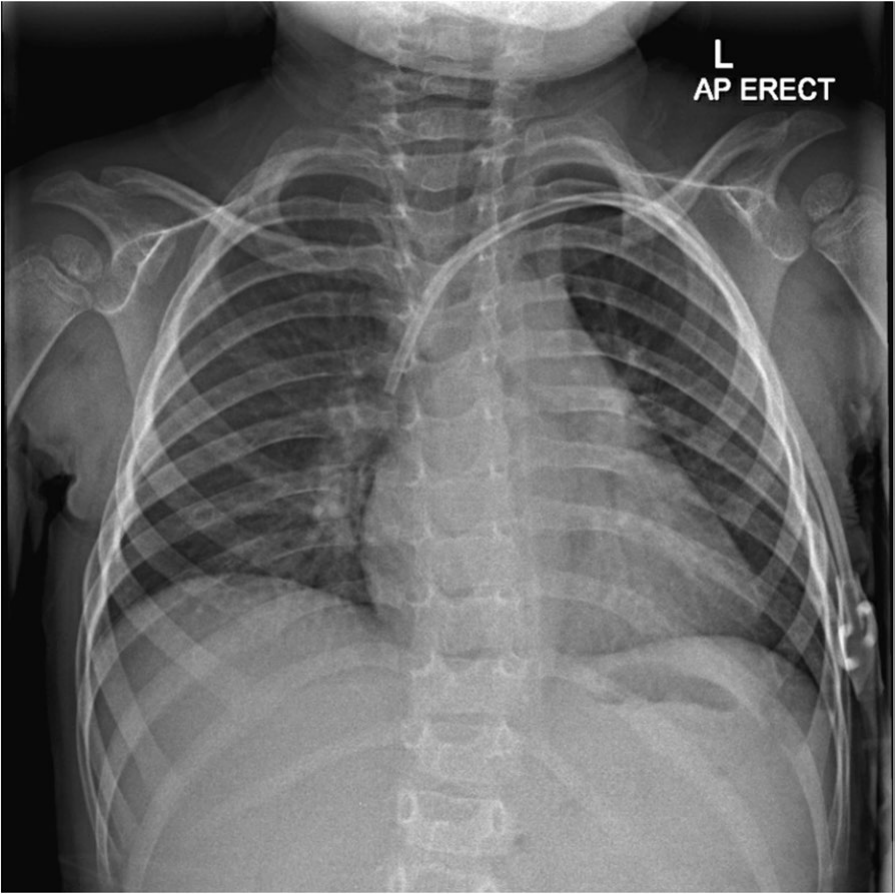
Preprocedural control image showing left sided dialysis line in situ with tip projected over the right atrium

Figure 1 shows control image with dialysis line appropriately in situ.

A 0.035 hydrophilic angled tip Terumo guidewire was inserted via the dialysis catheter and snared with 25 mm Amplatz gooseneck snare via right 12fr common femoral vein access. Guidewire pulled through right common femoral sheath. Penumbra Lightning 12™ mechanical thrombectomy device advanced into the right atrium over the through-and-through Terumo guidewire from the groin access.

**Fig. 2:**
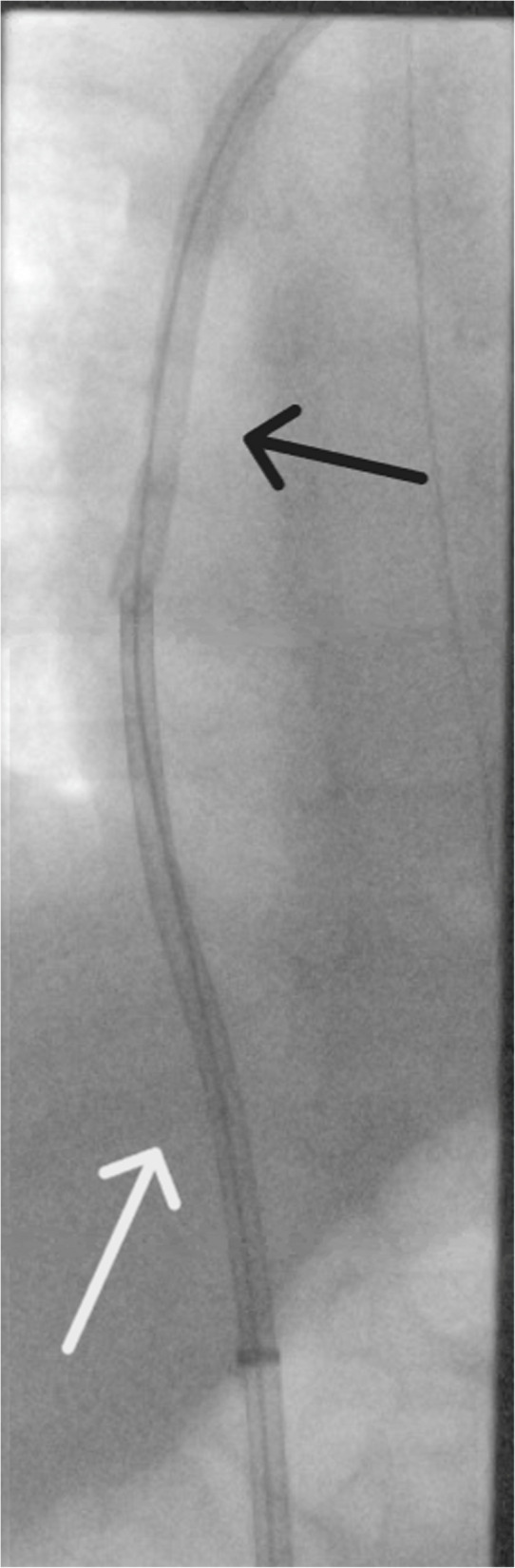
Intraprocedure fluoroscopic image demonstrating docking of penumbra thrombectomy device with dialysis catheter using through and through wire technique. The 12 French penumbra catheter (white arrow) is in the right atrium with tip against the dialysis catheter (black arrow) over an amplatz wire after a 0.035 hydrophilic angled tip terumo guidewire was inserted via the dialysis catheter and snared with 25 mm amplatz gooseneck snare via right 12fr common femoral vein access

Clot retrieval initially performed over the guidewire demonstrated in Fig. 2. This was followed by free aspiration in the right atrium. A mixture of clot and fibrin was aspirated. Following mechanical thrombectomy the dialysis catheter was removed from the left internal jugular vein.

**Fig. 3:**
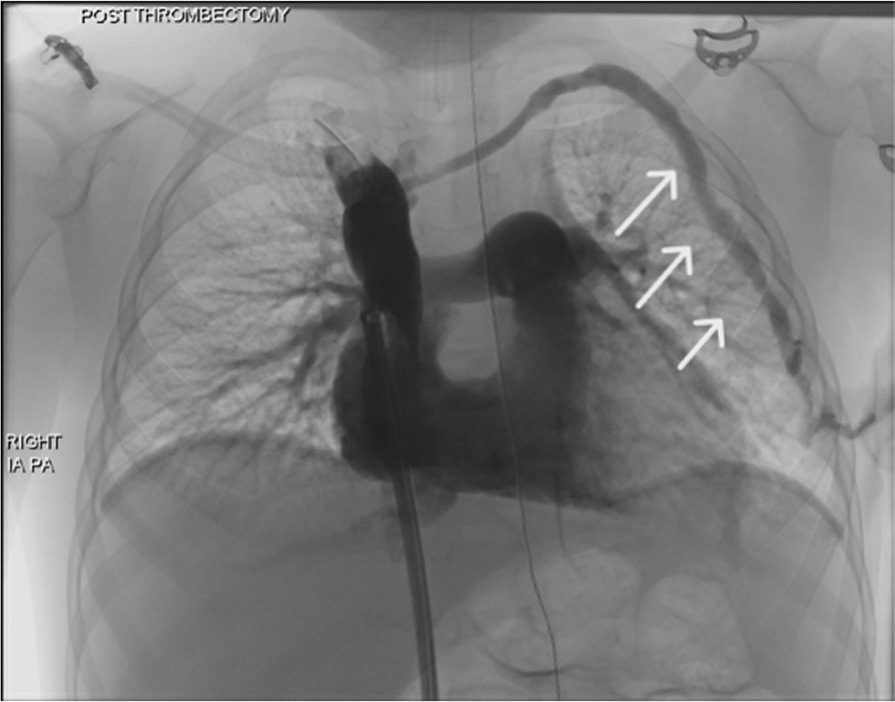
Post-thrombectomy fluoroscopic image demonstrating active contrast extravasation (white arrows) along the subcutaneous catheter tract, consistent with acute hemorrhage following dialysis line removal. This image, obtained immediately after catheter withdrawal, shows contrast tracking through the tissue planes from the left internal jugular venous entry site toward the anterior chest wall. Contrast delivered from 4 french MPA catheter within SVC

Completion angiography showed no evidence of embolisation to the pulmonary arteries; however, the operator saw haemorrhage both clinically from the chest access site and fluoroscopically along the tract as demonstrated in Fig. 3.

Prompt control was established with compression to the neck however haemostasis was not achieved after 30 min of manual compression. Although the patient was haemodynamically stable throughout, given the patient was anticoagulated and significant volume of aspiration during thrombectomy, the decision was made to embolise tract. 5 cc of human gelatine-thrombin matrix was administered to the tract with compression maintained at the neck to prevent reflux into the systemic venous system.

Along with aspiration from the thrombectomy device there was a combined blood loss of 300 ml. Intraprocedural Hb dropped to 79 g/l from 107 g/l and the decision was made for transfusion. A sample of clot and catheter tip was sent for microscopy, culture and sensitivities which later yielded staph. epidermidis. The patient was subsequently transferred to PICU where his Hb remained stable. He was treated with intravenous antibiotics and treatment dose dalteparin for a further 9 days with resolution of pyrexia and no clinical evidence of embolisation. Serial echocardiograms 3 and 7 days post procedure showed some residual thrombus which was thought to be residual fibrin cast from CVC. The patient was then lost to follow up as he and his family returned home outside the UK with a plan for review by cardiology at his local hospital.

## Discussion

This case describes a difficult clinical scenario without established evidence for best practice. Literature on paediatric mechanical thrombectomy is limited, with most applications extrapolated from evidence in adult populations [6, 7].

We show the importance of integrated MDT decision making involving nephrology, cardiology, and interventional radiology to make an individualised risk benefit analysis for the patient. In this case, an expanding clot despite optimised medical management and ongoing infection, as well as known low level evidence in adults favoured thrombectomy.

Technically, a through-and-through wire technique was used to minimise the risk of clot disruption and provide accurate stable application of the device. This was followed by free aspiration to remove any remaining clot after first pass.

We illustrate a rare but important complication of tract haemorrhage following the removal of the central venous catheter. Human gelatine-thrombin matrix (floseal) was used in this case for tract embolisation. It consists of a bovine-derived gelatin matrix and a human-derived thrombin component. The matrix acts as a physical barrier, while the thrombin facilitates the formation of a stable fibrin clot at the bleeding site. Alternative options would have included surgical closure using simple 2.0 non-absorbable suture to tract or retrograde access and plugging from groin sheath. However, this agent is ideal for filling an irregular subcutaneous tract and will stop bleeding even in the presence of anticoagulation as in this case [8]. Further advantages include the material is biodegradable over approximately 6 to 8 weeks and has been shown in other surgical settings to control bleeding faster than ligation or gelfoam [8]. Haemorrhage control is of particular importance in these cases with another case series finding post operative transfusion rate reaching up to 46% [9].

## Conclusions

We describe a rare combination of catheter related complications. We are unaware of any previous published examples of tract haemorrhage shown fluoroscopically. Although mechanical thrombectomy can be a valuable intervention for removing thrombi in the right atrium, particularly in context of infected clot, further good quality evidence is needed to support its use.

This case underscores the need for further research into paediatric-specific guidelines for thrombectomy and catheter management to minimise complications and improve patient outcomes.

## Data Availability

Data sharing is not applicable to this article as no datasets were generated or analysed during the current study.

## Abbreviations

CVC: Central venous catheter
MDT: Multidisciplinary team
